# An alternative supplemental feeding method for preterm infants: the supplemental feeding tube device

**DOI:** 10.3906/sag-2009-323

**Published:** 2021-08-30

**Authors:** Müjde ÇALIKUŞU İNCEKAR, Seda ÇAĞLAR, Fatma KAYA NARTER, Emriye TERCAN TARAKCI, Emine ÖZPINAR, Esra DEMİRCİ ECEVİT

**Affiliations:** 1 Department of Pediatric Nursing, Faculty of Health Sciences, Yüksek İhtisas University, Ankara Turkey; 2 Department of Pediatric Nursing, Florence Nightingale Faculty of Nursing, İstanbul University - Cerrahpaşa, İstanbul Turkey; 3 Neonatal Intensive Care Unit, Kartal Dr. Lütfi Kırdar City Hospital, İstanbul Turkey; 4 First and Emergency Aid Program, İstanbul Gedik University, İstanbul Turkey

**Keywords:** Bottle feeding, breastfeeding, supplemental feeding methods, neonatal intensive care unit, premature

## Abstract

**Background/aim:**

The purpose of this study was to determine the effects of the supplemental feeding tube device (SFTD) and bottle methods on weight gain, transition to full breastfeeding, breastfeeding success, and duration of discharge in preterm infants.

**Materials and methods:**

This randomized controlled trial was conducted with a total of 46 preterm infants including 23 infants in study (SFTD) and control (bottle) groups. An information form, an infant follow-up form for feeding, and LATCH breastfeeding assessment instrument were used to collect the data.

**Results:**

The gestation week of the infants in the study group was 31.22 ± 2.76, and in the control group it was 30.52 ± 2.47. The birth weight of the infants in the study group was 1586.3 ± 525.35 g and 1506.09 ± 454.77 g in the control group. The daily weight gain of the infants was 24.09 ± 15.21 g in the study group and 27.17 ± 17.63 g in the control group. The infants in the study group (4.70 ± 2.44 days) transitioned to full breastfeeding earlier than those in the control group (6.00 ± 4.10 days). LATCH 2nd measurement scores were significantly higher in both groups than LATCH 1st measurement scores (p < 0.01). Although it was not statistically significant (p > 0.05), the infants in the study group (10.22 ± 5.20 days) were discharged earlier than those in the control group (13.48 ± 8.78 days).

**Conclusion:**

The SFTD and bottle methods were determined to be similar in terms of daily weight gain, transition to full breastfeeding, breastfeeding success, and duration of hospitalization.

## 1. Introduction

Independent oral feeding is an important issue for preterm infants, since it predicts hospital length of stay [1,2]. Among infants with a stable cardiopulmonary status oral feeding is usually started at postmenstrual age of 33 to 34 weeks. During this period, their sucking pattern is similar to the pattern of term infants when the two components of sucking (rhythmic alternation of suction and expression) are considered [1].

Any therapy or instrument, improving preterm infants’ oral feeding skills, enables them to perform successful and safe oral feeding, reduces their length of hospital stay, accelerates the reunion between mother and infant, and reduces medical costs [2]. Alternative feeding devices include the use of bottles, supplemental feeding tube devices (SFTDs), finger feeding, cups, and syringes [3,4]. Many healthcare professionals and international board certified lactation consultants recommend supplemental feeding devices instead of the bottle [3]. The exclusive breast feeding rates and total breast feeding periods are not found to be in the expected level because of extensive bottle feeding [5]. Exclusive breastfeeding in full breastfeeding is defined as the baby not eating or drinking anything other than breast milk [6]. Breastfeeding is one of the most effective ways to ensure health and survival of an infant [5]. The World Health Organization (WHO) launched the “Baby Friendly Hospital Initiative” for support of breastfeeding and has published the “Ten Steps to Successful Breastfeeding” for facilities providing maternity and newborn services World Health Organization, UNICEF. Ten steps to successful breastfeeding; 2018.1. The 9th step of these steps is expressed as “Counsel mothers on the use and risks of feeding bottles, teats and pacifiers”. SFTD is presented as an alternative for bottle use. There are many “baby-friendly’ hospitals in the world and in Turkey [5]. In a systematic review investigating 58 studies on birth and neonatal care, a correlation was found between giving birth in a baby friendly hospital and improvement possibility of breastfeeding outcomes and it was obviously indicated that adhering to “Ten Steps to Successful Breastfeeding” affected the breastfeeding rates [7].

In a previous study, it was reported that the bottle feeding rarely maintained a breastfeeding relationship in 5 geographic regions (Asia, Australia, Canada, South America, and the USA), and although it was rarely preferred, it was the most commonly used feeding method. The majority of the respondents reported that SFTD best preserved the breastfeeding relationship and it was preferred as reinforcement method [3].

Bottle feeding with artificial milk requires less work and the baby reaches milk more easily. Nevertheless, this intervention does not give the infant access to the beneficial elements of human milk and does not provide the breastfeeding bond desired by the mother [8]. SFTD refers to a tool used for supplemental nourishment of the infant during breastfeeding. This tool has a container containing human milk or artificial milk, being held by the mother or hung around the mother’s neck. A thin tube is attached to the mother’s breast using a tape extending slightly towards sides of the nipple [8].

Many lactation consultants have advocated the use of SFTD to maintain a breastfeeding relationship and to feed the infant in the breast [8]. Although there is insufficient number of evidence about the use of SFTD [3], the American Academy of Family Physicians, the United States Breastfeeding Committee, and many health departments like the States of Indiana and California suggest the use of supplemental feeding tube devices to supplement breastfeeding [4].

Currently, there are three commercially available SFTDs: Supplemental Nursing System (SNS) (Medela AC, Baar, Switzerland), the Lact-Aid Nursing Training System (Lact-Aid International, Inc., Ponte Vedre Beach, USA), and the Jack Newman Lactation Aid and Feeding Tube (Lactation Connection, Bluff Dale, Texas) [4]. Despite suggestions for its use, evidences supporting the use of these devices have not been well described, yet [4,8]. Since the SFTD method provides skin-to-skin contact of the mother with her infant and allows him/her to suck the breast compared to the bottle feeding method, it may also have an effect on the bonding between the mother and her infant and human milk production. The purpose of this study was to determine the effects of the SFTD and bottle methods on weight gain, transition to full breastfeeding, breastfeeding success, and time between transition to full breastfeeding and discharge in preterm infants.

## 2. Materials and methods

### 2.1. Study design

This randomized controlled trial was conducted at the level III neonatal intensive care unit (NICU) with 30 beds in a tertiary hospital with 750 beds in İstanbul between August 2016 and September 2017. The hospital included lactation counselling unit. Moreover, since it was a baby friendly hospital, a training including 16-h theory and 4-h practice was provided to all nurses working in the hospital. The content of the training included the subjects related to correct practices of breastfeeding such as the condition of breastfeeding, importance of human milk, physiology of breastfeeding, problems related to breastfeeding, baby friendly hospitals, communication, and counselling.

### 2.2. Participants

**Figure 1:**
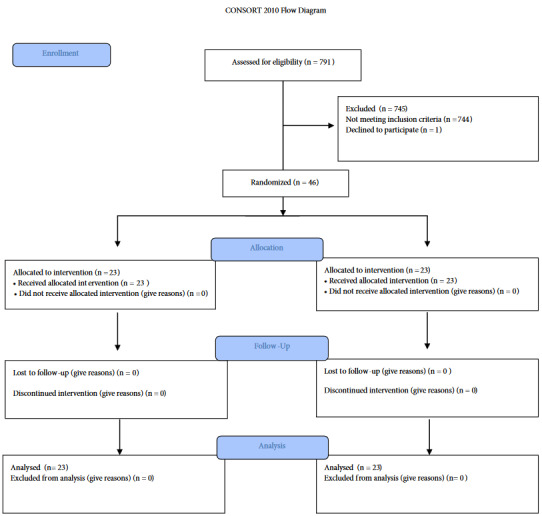
CONSORT flow diagram.

A power analysis was performed in this study using the LATCH Breastfeeding Assessment Tool [9]. The effect size was 1.09, the power was 0.95, ß was 0.05, and α was 0.05. The sample size was determined as a total of 46 preterm infants including minimum 23 infants for each group. In the CONSORT diagram [10], the groups were shown in Figure 1. Randomization was performed by randomly distributing the numbers 1**–**46 into two groups via a computer program without repetition. 

The inclusion criteria of the study were determined as follows: being at the postmenstrual age (PMA) of 34 weeks; being ≤ 1250 g, having previous enteral feeding via orogastric tubes, having gavage-based feeding with the human milk, and the mother’s willingness to breastfeed their infant; based on the cue-based feeding approach to readiness for feeding [11] tolerating enteral feeding, having a stable oxygen saturation and respiratory system during feeding, having the ability to lick, nuzzle or suck nonnutritive, being able to transit to the alert state, and rooting in response to touch around the mouth and lips. The exclusion criteria of the study included having congenital anomaly, sepsis, chromosomal disorder, and intracranial hemorrhage.

### 2.3. Setting

#### 2.3.1. The infant-mother information and follow-up forms 

The form included the items about the infant’s sex, birth weight, birth length, and weight during the first oral feeding, feeding frequency, and weight gain follow-up as well as mother’s age, education level, mode of delivery, number of living children, and breastfeeding experience.

#### 2.3.2. LATCH breastfeeding assessment instrument

Breastfeeding success of the mothers was assessed by using LATCH. The instrument was developed by Jensen et al. [12] to evaluate mothers’ breastfeeding success. This assessment instrument is comprised of 5 assessment criteria: latch (L), audible swallowing (A), type of nipple (T), comfort [breast/nipple] (C), and hold [positioning] (H). Each item is rated between 0 and 2 and total score is 10 points. A high score signifies successful breastfeeding [12]. This scale was adapted into Turkish by Yenal and Okumuş [12]. The Cronbach’s alpha coefficient of the scale was 0.95 [13]. In this study, the intraclass correlation coefficient (ICC) of the scale was found to be 0.81 LATCH 1st measurement and 0.77 LATCH 2nd measurement. LATCH was routinely being used in the clinic. Latch assessment was recorded by two nurses independently and simultaneously during the study.

#### 2.3.3. Supplemental nursing system and bottle

In this study, the SNS was used for feeding infants in the study group. The device has two probes enabling the mother to breastfeed (multiple infants) at the same time from both breasts. Two tubes in the device for right and left breasts were attached to the mother’s nipple. When the mother breastfed her infant with a probe of the device fixed to her breast, the other probe of the device was clamped. The SNS is a sterile product with an adjustable human milk flow system and an adjustable neck strap. It is produced without bisphenol A (BPA) and all of its parts are directly contact with the human milk. The bottle, which has a narrow mouth and is sterile and latex-free was used for the infants in the control group. The feeding bottle expands by means of suction-free air-duct feature, thus allowing the preterm infant to easily suck with low pressure.

### 2.4. Procedure

In the unit, preterm infants were fed with the bottle method. The SFTD method was a new method for the unit in the supplemental feeding method. Therefore, the SFTD method was determined as the study group and the bottle method was determined as the control group. The study was conducted in an empty, quiet room where mothers breastfeed their infants comfortably between 8:00 am and 4:00 pm on weekdays, at 10:00 am, 1:00 pm and 4:00 pm feeding hours. In the first stage of the study, a breastfeeding consultant nurse of the unit provided the mothers in both groups with training on breastfeeding. The content of the training included the subjects of importance of human milk, feeding with breastfeeding, increasing techniques of feeding with human milk, and providing feeding with quality human milk.

**Figure 2:**
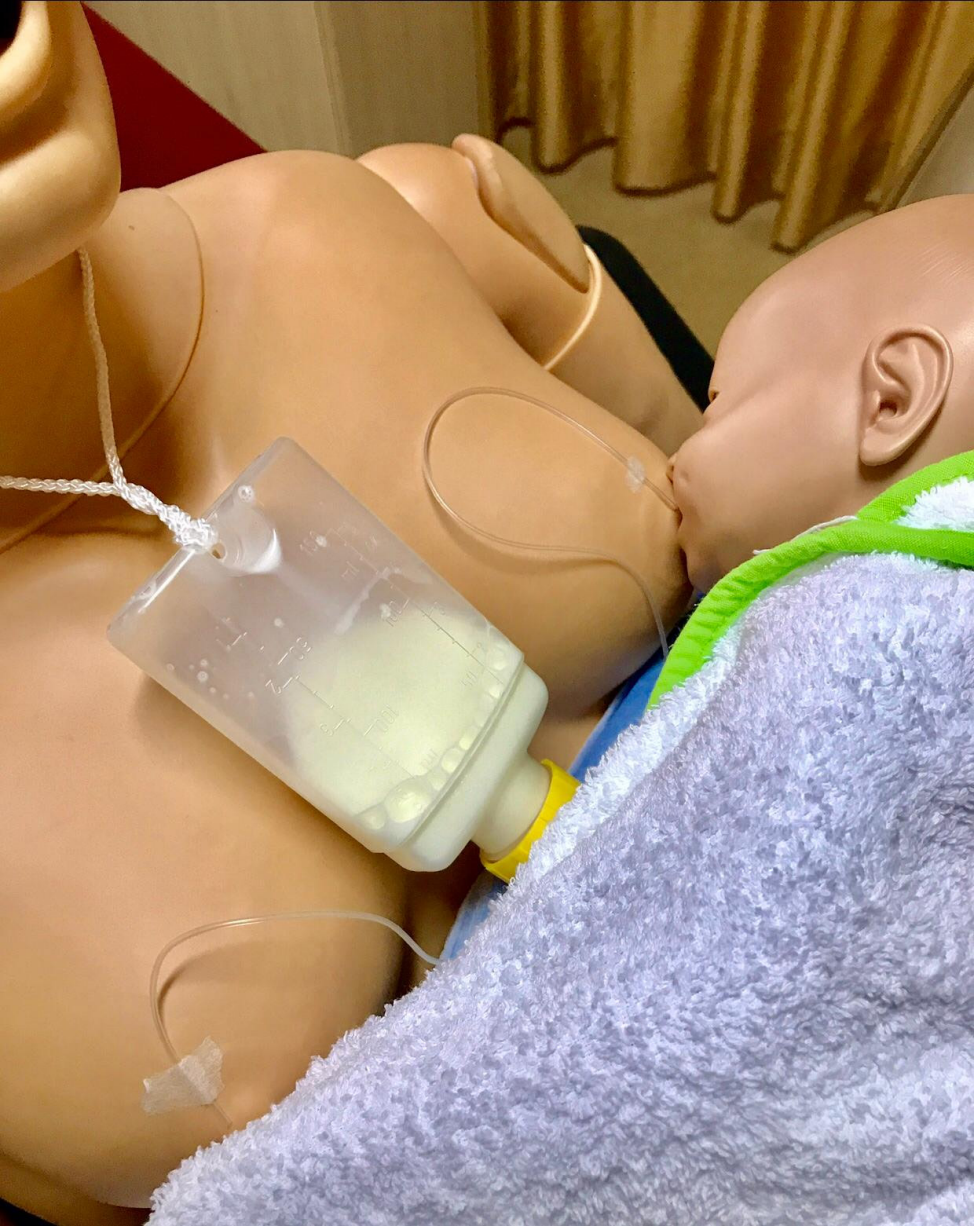
The study group.

**Figure 3:**
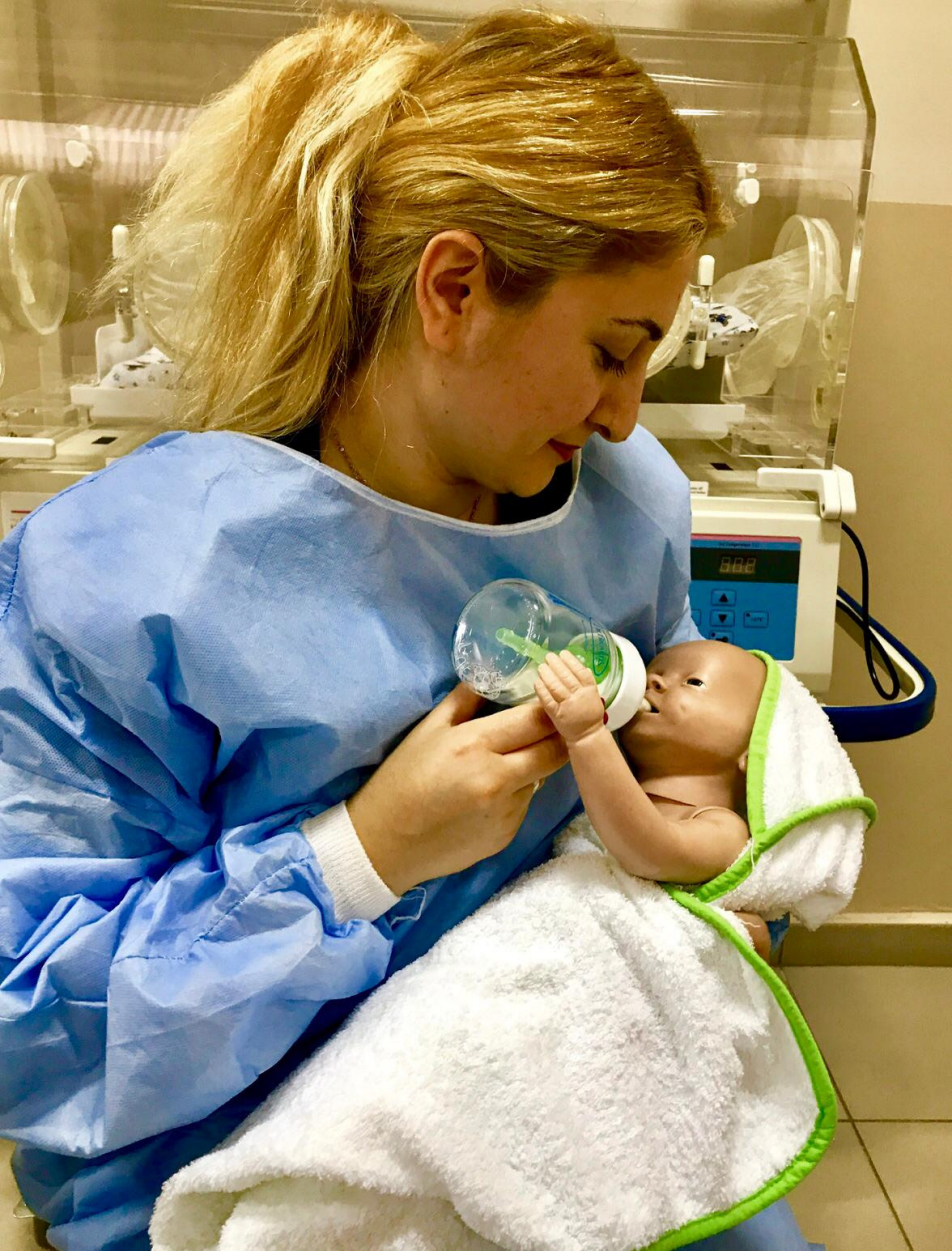
The control group.

During the initial breastfeeding, all the mothers’ breastfeeding was independently assessed by two nurses in the research team at the same time (LATCH, measurement 1). Due to the fact that the preterm infants in the study and control groups experienced sucking the breast of their mothers and skin-to-skin contact was achieved, mothers tried to breastfeed their infants for the first 10 min. The mothers in the study group breastfed their infants for 20 min using the SNS device (Figure 2). The preterm infants in the control group were fed by their mothers for 20 min with a bottle containing human milk in their mothers’ arms and in the breastfeeding position (Figure 3). The preterm infants in the study and control groups were fed with human milk using orogastric tube between 4:00 pm and 8:00 am and at all feeding times on weekends. Until all preterm infants’ transition to full breastfeeding is achieved, 4.4 g human milk fortifier was added to 100 mL human milk. The human milk fortification product used in our NICU is Eoprotin (Milupa AG, Friedrichsdorf, Germany) 4.4 g of which contain 1.1 g protein and 15 kcal energy. Weights of all preterm infants were measured and recorded at 8:00 am every day until they were transition to full breastfeeding.

The preterm infants in the study and control groups were breastfed by their mothers between 8 am and 12 midnight on weekdays and weekends after transition to full breastfeeding. All preterm infants were fed with human milk using an orogastric tube from 1:00 am to 8:00 am. During this period, weights of all infants were measured and recorded every day at 8:00 until discharge. At the last breastfeeding of all preterm infants before discharge, in order to evaluate the breastfeeding success of their mothers, breastfeeding of the mothers was independently and simultaneously evaluated by two nurses in the research team using a measuring tool (LATCH, measurement 2).

### 2.5. Ethical considerations

The ethical approval from the Clinical Trials Ethics Committee of the Kartal Dr. Lütfi Kırdar Training and Research Hospital (IRB: 2016/514/86/4) and institutional permission from the same hospital were obtained. While informed written consent from the families were obtained, written permission was received from the author of the scale via-email for the use of the scale.

### 2.6. Data analysis

The IBM SPSS Statistics v: 22.0 (IBM Corp., SPSS, Turkey) was used for statistical analysis of the results of the study. The Shapiro–Wilks test was used to evaluate the compatibility of the variables to normal distribution. The data of the study were evaluated using descriptive statistical methods (percentage, mean, standard deviation, median). The student t-test for assessment of the normally distributed quantitative data between two groups and the Mann–Whitney U test for assessment of nonnormally distributed quantitative data were used. The Wilcoxon signed ranks test was used to evaluate the scale scores before and after STFD in study group. The intraclass correlation coefficient (ICC) was used to assess the interobserver agreement. The Chi-square and Fisher’s exact tests were used to assess the qualitative data. Significance was evaluated at the level of p < 0.05.

## 3. Results

### 3.1. Descriptive characteristics

There was not any significant difference between both groups in terms of the descriptive characteristics of the infants and their mothers (p > 0.05) (Table 1).

**Table 1:** Characteristics of infants and mothers (n = 46).

Preterm infants			Study group (n = 23)	Control group (n = 23)	p
			Median (min-max)	Median (min-max)	
Birth weight (g)			1730 (600–2480)	1585 (680–2200)	10.560
Birth length (cm)			40 (30–49)	40 (31–45)	10.965
Weight in the first oral feeding			1750 (1300–2450)	1705 (1330–2670)	10.684
			n (%)	n (%)	p
Sex	Female		9 (39.1)	11 (47.8)	30.767
	Male		14 (60.9)	12 (52.2)	
Mothers			Median (min-max)	Median (min-max)	p
Age (year)			28 (18–44)	30 (20–41)	20.259
Gravida			2 (1–4)	2 (1–5)	10.966
Previous experience breastfeeding (month)			14 (2–30)	12 (1–24)	10.334
			n (%)	n (%)	p
Level of education		Elementary	12 (52.2)	14 (60.9)	30.766
		≥ High school	11 (47.8)	9 (39.1)	

Note = The values are presented as median (min-max).1Z: Mann–Whitney U test,2Student t test.χ2: Chi-square and Fisher’s exact Chi-square tests.

### 3.2. Weight gain, duration of full breastfeeding, breastfeeding success, and time between transition to full breastfeeding and discharge

The daily weight gain of the infants was 24.09 ± 15.21 g in the study group and 27.17 ± 17.63 g in the control group. No significant statistical difference was found between the groups in terms of weight gain (p > 0.05). The infants in the study group (4.70 ± 2.44 days) transitioned to full breastfeeding earlier than the infants in the control group (6.00 ± 4.10 days). No significant statistical difference was found between the groups in terms of transition time to full breastfeeding (p > 0.05). The second LATCH measurement scores were significantly higher in both groups than 1st LATCH measurement scores (p < 0.01). The infants in the study group (10.22 ± 5.20 days) were discharged earlier than those in the control group (13.48 ± 8.78 days). No significant statistical difference was found between the groups in terms of discharge period (p > 0.05) (Table 2).

**Table 2:** Distribution of infants with weight gain, full breastfeeding, breastfeeding success, and duration of discharge (n = 46).

Feeding methods	Study group (n = 23)	Control group (n = 23)	p
	Median (min-max)	Median (min-max)	
Weight gain (day)	23 (3–54)	24 (1–65)	10.605
Transition to full breastfeeding (day)	4 (1–11)	4 (1–14)	10.490
Breastfeeding success (LATCH)			
LATCH 1st measurement score	8 (6–10)	8 (6–9)	0.135
LATCH 2nd measurement score	10 (9–10)	10 (8–10)	0.413
p	40.001*	40.001*	
Time between transition to full breastfeeding and discharge (day)	10 (2–27)	11 (2–38)	10.321

Note = The values are presented as median (min-max).1Z: Mann–Whitney U test,4Z: Wilcoxon signed ranks test.

## 4. Discussion

A preterm infant’s poor sucking capability and irregular sucking rhythm may discourage the mother from breastfeeding. Hence, mothers may require support and breastfeeding counselling to breastfeed and/or provide human milk during this period [15,16]. It may be necessary to support both the newborn, who struggles with grasping the nipple, and the mother, utilizing the necessary means (i.e. supplementary feeding/supplementation tools), in order to ensure a compatible breastfeeding period [16]. Breastfeeding, then, is primarily recommended when the preterm infants are thought to be ready to be fed orally WHO/UNICEF/USAID. Indicators for assessing infant and young child feeding practices. Geneva, Switzerland: World Health Organization; 2008.2. However, given that preterm infants are generally unsuccessful at terms of sucking and taking the human milk during their initial breastfeeding experienced in their mothers’ breast, it is required to support sucking behaviors with different feeding methods [17].

Cups, bottles, syringes, finger feeding, and the use of SFTDs are recommended as supplementary feeding methods in the literature [4]. The bottle method is not recommended since it reduces the intake of human milk in the long term [18,19], even though this method was used in some previous studies [9,20–23]. Based on the results of the 2018 Turkey Demographic and Health Survey, it was found that 59% of infants aged between 0–1 months were fed exclusively with human milk Hacettepe University Institute of Population Studies. The 2018 Turkey demographic and health survey; 2018.3. This rate decreased to 10% in infants aged between 4–5 months due to the use of bottle [24]. It has been reported that the SFTDs can help protect the breastfeeding relationship and contribute to the WHO’s goal of increasing specific breastfeeding rates [3].

The infants in the study group transitioned to full breastfeeding within a shorter time and were discharged from the hospital earlier; however, their weight gain was less than the control group. Higher weight gain in the control group may be associated with the fact that it is easier for the infants to access the human milk from the bottle and they can also suck it more. In a randomized controlled trial conducted in Australia, it was reported that the novel feeding system (SFTD) and bottle fed groups were similar in terms of the period of transition to full breastfeeding and those in the novel system group stayed in the hospital for a shorter time [25]. In a systematic review, SFTD method was found to be beneficial for breastfeeding mothers as a supporting breastfeeding method [4]. 

In this sense, the strength of the present study is that all of the infants reaching full breastfeeding were breastfed by their mothers and thus discharged. 91.3% (n = 21) of the mothers in the study group were satisfied with the SFTD method. On the other hand, two mothers were unsatisfied with the device since they thought that the device only made breastfeeding more difficult, the rate of flow was high, and the probe tip was stiff. In a study conducted in America, the opinions of 22 mothers who used SFTDs, were taken. It was reported that many mothers liked the device, but some others did not; likewise, several stated that even though they disliked the device, it helped them. It was determined that the mothers liked the device because the SFTD allowed them to successfully breastfeed their infants, to continue breastfeeding, and to have the desired breastfeeding relationships. The mothers who disliked the device described it as “bulky”, “time-consuming”, “artificial”, “complex”, “difficult to use”, and “untidy”. In conclusion, it was reported that SFTD was an acceptable valuable alternative in terms of helping mothers achieve their breastfeeding goals [8]. 

The fact that majority of the mothers using SFTD were satisfied with the method was a very important motivation for the present study. This method moreover helped mothers and their infants to spend 30 min together in a safe environment, which in turn resulted in skin-to-skin contact between the mother and infant via the breastfeeding.

### 4.1. Study limitations

Limitation of the study was that SFTD was applied only between 8:00 am and 4:00 pm on weekdays. Therefore, the infants were given human milk via an orogastric tube between 4:00 pm and 8:00 am. In addition to, after transition to full breastfeeding, the mothers were in the hospital between 8:00 am and 12:00 midnight. These limitations may have an effect on the preterm infant’s transition to full breastfeeding and the duration of hospitalization. Although the infants in the SFTD group had skin to skin contact with their mothers for 30 min and the infants in the bottle group for 10 min, the effect of skin-to-skin contact was not evaluated due to the time difference between the groups.

## 5. Conclusion

The SFTD and bottle feeding methods were determined to be similar in terms of daily weight gain, transition to full breastfeeding, breastfeeding success, and duration of hospitalization. The SFTD method can be used as an alternative method in the process of transition to full breastfeeding of preterm infants. It was concluded that the SFTD was an effective feeding method among systems supporting the breastfeeding for feeding of the preterm infants in NICU and the mothers were satisfied with this method. It is important for lactation consultants, nurses and caregivers to have the knowledge and skills for providing the best supplemental feeding method to a mother and her infant in order to maintain a breastfeeding bonding. It may be recommended to conduct future studies with large sample groups to compare SFTD and other feeding methods and to examine with parameters such as physiological variables, anthropometric measurements, feeding tolerance, and bonding during the transition of the preterm infant to full breastfeeding.

## Funding

The authors received no financial support for the research, authorship, and/or publication of this article.

## Informed consent

The ethical approval from the Clinical Trials Ethics Committee of the Kartal Dr. Lütfi Kırdar Training and Research Hospital (IRB: 2016/514/86/4) and institutional permission from the same hospital were obtained. While informed written consent from the families were obtained, written permission was received from the author of the scale via email for the use of the scale.
